# Sedation with propofol during ERCP: is the combination with esketamine more effective and safer than with alfentanil? Study protocol for a randomized controlled trial

**DOI:** 10.1186/s13063-017-2197-8

**Published:** 2017-10-11

**Authors:** Susanne Eberl, Lena Koers, Jeanin E. van Hooft, Edwin de Jong, Thomas Schneider, Markus W. Hollmann, Benedikt Preckel

**Affiliations:** 1 Department of Anesthesiology, Academic Medical Centre, University of Amsterdam, Meibergdreef 9, Amsterdam, 1100 DD The Netherlands; 2 Department of Gastroenterology & Hepatology, Academic Medical Centre, University of Amsterdam, Meibergdreef 9, Amsterdam, 1100 DD The Netherlands; 3 Department of Anesthesiology, Tjongerschans Ziekenhuis, Thialfweg 44, 8441 PW Heerenveen, The Netherlands

**Keywords:** Procedural sedation, Propofol, ERCP, Esketamine

## Abstract

**Background:**

Endoscopic retrograde cholangiopancreatography (ERCP) is a gastrointestinal procedure that requires a relatively motionless patient during the intervention. Deep sedation by intravenous propofol combined with an opioid has recently become the preferred sedation technique. However, when high doses of propofol are used, side effects, namely respiratory depression, may occur. Esketamine has hypnotic, analgesic, and sympathomimetic effects. Our assumption is that a combination of propofol with esketamine reduces the dosage of individual drugs, thereby minimizing sedation side effects while keeping the same satisfaction level of patients and endoscopists.

**Methods/design:**

The study will be performed as a randomized controlled multicenter trial. Patients undergoing ERCP, ≥ 18 years old, with American Society of Anesthesiologists (ASA) classification I–III will be randomized after written informed consent to group K (propofol/esketamine) or to group A (propofol/alfentanil). The primary outcome, reflecting effectiveness of sedation, is the total dose of propofol. Secondary outcome parameters are patients’ and endoscopists’ satisfaction with the procedure and the number of sedation-related pulmonary and cardiovascular events. Data on sedation-related events are collected by recording of oxygen saturation (SpO_2_), respiratory rate (RR), end-tidal CO_2_ (etCO_2_), heart rate (HR), arrhythmias (electrocardiogram (ECG)), and non-invasive blood pressure (NIBP) measurements. Satisfaction parameters are collected by means of questionnaires before and after the procedure and on the following day.

**Discussion:**

Esketamine is known for its effective anesthetic and analgesic effects maintaining spontaneous breathing and airway reflexes. Due to an increase in sympathetic tone, hypotension and cardiac depression is less common. Unfortunately esketamine is also known for its psychotomimetic effects. We aim to demonstrate that the combination of esketamine with propofol for sedation in patients subjected to ERCP interventions is nevertheless superior to a combination of propofol with an opioid.

**Trial registration:**

Nederland’s Trial Register, NTR5486. Registered on 17 September 2015.

**Electronic supplementary material:**

The online version of this article (doi:10.1186/s13063-017-2197-8) contains supplementary material, which is available to authorized users.

## Background

Endoscopic retrograde cholangiopancreatography (ERCP) is a complicated, often painful gastrointestinal procedure. It is used for diagnostic purposes in biliary and pancreatic disease and also for therapeutic interventions such as sphincterotomy, gallstone extraction, and biliary and pancreatic duct stenting. Because any movement of the patient could importantly affect success of the ERCP, procedures are usually performed under deep sedation or even general anesthesia with the patients in the semi-prone or prone position [1–3]. Over the last decade the combination of propofol and an opioid has become the preferred sedative regime during ERCP in many countries, despite known side effects.

Esketamine, the s-enantiomer of ketamine - is not only a well-known sedative, but also has strong analgesic properties. Furthermore, its sympathomimetic qualities can counteract the hemodynamic depression of propofol and so reduce the risk of cardiovascular and respiratory depression during sedation. A potential problem of esketamine could be its psychotomimetic effects, such as visual disturbances, vertigo, or nausea that could compromise patient satisfaction. There is still little evidence of an improved safety profile of a combination of propofol/esketamine and it is still open to discussion whether esketamine psychomimetic effects play a significant role in outpatient treatment.

## Methods/design

### Aim of the study

We hypothesize that procedural sedation with propofol and esketamine reduces the dosage of individual drugs, thereby minimizing sedation side effects while keeping the same satisfaction level of patients and endoscopists. To test this hypothesis we compare two groups. Group K receives propofol/esketamine sedation; group A receives propofol/alfentanil sedation during ERCP. Both groups receive standard deep sedation with propofol target controlled infusion (TCI) provided by specialized sedation anesthesia nurses.

### Trial design

The study is designed as a prospective, randomized, controlled, multicenter trial and reported following the Standard Protocol Items: Recommendations for Interventional Trials (SPIRIT) statement [4]. The sponsor of this trial is the Department of Anesthesiology of the Academic Medical Center (AMC) in Amsterdam. The sponsor is responsible for the collection, management, analysis, and interpretation of data; writing of the report; and the decision to submit the report for publication. The study is supported by institutional funding. Additional file 1 shows the SPIRIT checklist that we followed in this report.

### Participants

#### Number of patients needed

Alfentanil reduces the required hypnotic dose of propofol by 20–50% at fairly low doses according to the literature [5]. A 15% reduction in propofol requirement when using esketamine instead of alfentanil would reflect a total dose reduction of propofol by up to 65%, which is not only a statistically significant but also a clinically relevant difference.

The sample size calculation is based on retrospectively obtained observational data from previous ERCPs, collected in our hospital sedation database. The primary endpoint is the difference in the dose of propofol used. The mean dosage of propofol during ERCP was 580 mg, with a standard deviation of 190 mg. We will need to study 76 subjects in each group, given power of 0.80 and type I error of 0.05, to reduce propofol requirement by about 15%. Considering a dropout rate of 10%, the estimated sample size will be 83 patients per group, thus a total of 166 patients will be randomized.

#### Eligibility

The study takes place at two centers: the Department of Gastroenterology and Hepatology in the AMC of the University of Amsterdam and the Department of Gastroenterology and Hepatology in the Tjongerschans ziekenhuis, Heerenveen, The Netherlands - beginning December 2015 to January 2018. Eligible patients for participation in this clinical trial are those planned to undergo elective ERCP under deep propofol sedation, aged above 18 years, and American Society of Anesthesiologists (ASA) classification I–III, who give written informed consent.

#### Exclusion criteria

Patients are excluded if the following criteria in the patients’ medical history are applicable:

- Age range < 18 years
- ASA classification IV or V
- Allergic reaction to planned medication
- History of unregulated or malignant hypertension
- Significant ischemic heart disease
- History of psychological problems or psychiatric disease
- Use of drugs that affect the central nervous system
- Substance abuse
- Chronic pain
- Pregnancy
- Seizure disorders
- Increased intracranial pressure

**Fig. 1:**
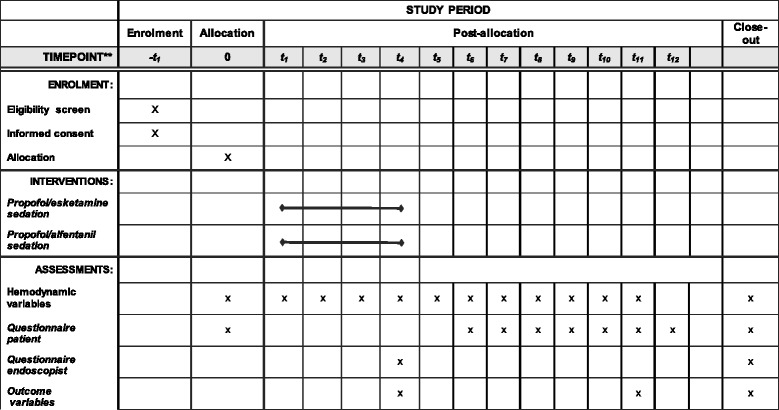
Schedule of enrollment, intervention, and assessment according to the Standard Protocol Items: Recommendations for Interventional Trials (SPIRIT) statement

The schedule of enrollment, intervention, and assessment is reported according to the SPIRIT statement (Fig. 1).

The number of excluded patients and the reasons for their exclusion will be reported according to the SPIRIT statement.

#### Consent

Patients’ medical history and their current state of health are screened on paper during standard anesthetic pre-assessment before the scheduled sedation. The investigator uses the anesthetic pre-assessment form to screen patients for inclusion and exclusion criteria. Patients meeting inclusion criteria are contacted by phone to verify criteria and asked for their willingness to participate in this study. Further information is sent by mail if they agree to participate. Final inclusion occurs after written informed consent is provided on the day of the procedure.

If patients decline to take part in the study, they are sedated according to the AMC standards with propofol and alfentanil. The investigator or physician performing the examination can decide to withdraw a subject from the study for urgent medical reasons (allergic reactions or acute health problems).

### Randomization

Patients are randomized online in both centers after signing informed consent using the ALEA software program provided by the Clinical Research Unit (CRU) of the AMC for centralized randomization in clinical trials. Patients are allocated to a treatment arm after the anesthetic nurse has entered patient details and absence of exclusion criteria in the ALEA program.

The study is performed as a single-blinded study. Because of safety reasons the anesthetic nurse is not be blinded to the treatment arm and will therefore perform the randomization in the ALEA program. The patient, endoscopist, and investigator are blinded to the allocated treatment arm.

Patient data are collected on case report forms (CRFs) in each center. Data processing will take place in the AMC using the Castor database and will be performed by the investigator or study coordinator. The CRU also will independently monitor both locations of this multicenter trial (AMC and Tjongerschans ziekenhuis) three times using duplicated measurements documented in the hospital data management systems with complete access to all databases.

### Intervention

All patients are fasted at least 6 hours before ERCP. Antibiotic prophylaxis is given according to hospital standards. As a standard procedure, diclofenac 100 mg is administered rectally immediately before the procedure to reduce post ERCP pancreatitis [6]. Procedural sedation is performed by anesthesia nurses trained in the standards of care for procedural sedation and analgesia (PSA) according to the Dutch national guidelines. An anesthesiologist is available for liaison, supervision and emergency help. Insufflation of the duodenum during ERCP is with CO_2_ instead of air, to reduce peri-procedural pain and abdominal distension.

Patients are asked to complete a questionnaire before the procedure to assess baseline pain, drowsiness, nausea, perception, and mood using a visual analog scale (VAS) (0 = 100). Baseline assessments of the Modified Observer’s Assessment of Alertness/Sedation Scale (MOAA/S), the Aldrete recovery score and measurements of heart rate (HR), non-invasive blood pressure (NIBP), respiratory rate (RR), and oxygen saturation (SpO_2_) are recorded.

After placement of an intravenous line, an infusion of 500 ml NaCl 0.9% is started at the rate of 250 ml/h. Five minutes before insertion of the endoscope, glycopyrrolate 0.2 mg, and lidocaine 50 mg are administered intravenously. Then patients are asked to place themselves into the prone or semi-prone position. From the start of sedation till the end of the endoscopic procedure, 2 L/min of oxygen are administered by nasal cannula, and HR, SpO_2_, RR, electrocardiogram (ECG), NIBP, end-tidal carbon dioxide (etCO_2_) and sedation level measured by the MOAAS/S are collected at 5-minute intervals. An independent, blinded observer collects research data.

Both groups are sedated by a propofol TCI system (Propofol 1% MCT Fresenius). TCI means a weight pre-programmed system using the Marsh pharmacokinetic model to attain a specific estimated propofol plasma target level [7]. We start propofol TCI in both groups with a targeted plasma level of 1.5 μg/ml. Reaching this plasma level, group K is treated with esketamine (Ketanest S, Pfizer) 150 μg/kg and group A is treated with alfentanil (Rapifen, Janssen-Cilag) 2.0 μg/kg. After 2 min propofol TCI is stepped up - if needed - to a maximum targeted plasma level of 2.5 μg/ml.

Before starting the endoscopic procedure, patients are assessed for their level of sedation using the MOAA/S scale yielding at a score <2. The modified form of the MOAA/S scale uses not only the responsiveness component of the original scale (awake (5) - unresponsive (1)) but is extended with assessment of painful stimuli. As reaction to painful stimuli are still possible at anesthetic levels that block reactions to verbal commands, prodding, or shaking they can be used to assess deeper sedation levels.

If MOAA/S is > 2, e.g. the patient is too responsive to tolerate the procedure; additional sedation is provided with TCI increments of 0.5 μg/ml plasma target level. These very small steps are performed in order to avoid deep sedation. For every step up of propofol TCI, additional esketamine 50 μg/kg or alfentanil 1 μg/kg is added. Maximum dosage is 500 μg/kg ketamine or 7.5 μg/kg alfentanil.

Total dosage of propofol, alfentanil, and esketamine and the time of the total procedure and length of time between the end of the procedure (removing the scope) till reaching an MOAAS score > 4 and the declaration that the patient is ready for transport to the recovery unit, is noted.

At arrival in the recovery room, monitoring is limited to SpO_2_, RR, ECG, and NIBP only. Recovery from anesthesia and the return of psychotomotoric fitness is assessed using the modified Aldrete score at arrival and 15, 30, 45, and 60 min later. This score describes the patient’s motoric activity, mechanical respiratory function, oxygen saturation, blood pressure, and consciousness and is designed to assess patient recovery after sedation. The total score is 10 [8]. During the time in the recovery room patients have to complete the identical questionnaire they completed at baseline, on pain, drowsiness, nausea, perception, and mood, using a VAS (0 = 100). Following daily standards, post-procedural pain is indicated as VAS > 40 and is treated with 2 mg morphine intravenously; nausea indicated by a VAS > 40 will be treated with 4 mg ondansetron intravenously.

Patients have to stay for at least 1 h in the recovery room. “Ready for discharge” is declared when an Aldrete score ≥ 9 or similar to the pre-procedural score is reached. The patient must be wide awake without suffering from side effects (e.g. nausea, dizziness), with stable hemodynamic signs, and able to ambulate without assistance. A follow-up telephone call will take place on the next day after the procedure.

### Primary objective

#### Definition of primary endpoint

The primary endpoint of the study - reflecting the effectiveness of coadministration of propofol and esketamine - is the total dose of propofol used.

#### Assessment of primary endpoint

We will record the total amount of propofol, esketamine, alfentanil, and all other drugs administered.

### Secondary objectives

#### Definition of secondary endpoints

Secondary endpoints focus on the satisfaction of patients and endoscopists with sedation and side effects, and on hemodynamic stability and safety, which is reflected in the number of respiratory and cardiovascular events.

#### Assessment of secondary endpoint

Pain, sedation level, and side effects such as nausea and psychotomimetic effects are recorded on questionnaires that patients have to fill in before and after the procedure. To assess post-procedural satisfaction patients are contacted the day after the procedure by telephone. In addition, endoscopists’ experiences with sedation are recorded on a questionnaire after the procedure.

#### Questionnaires

Before ERCP, after the procedure and arrival on the recovery unit, and on the following day patients are asked to complete questions on pain levels, drowsiness, nausea, perception, and mood using VAS scales (0 = 100). Pain intensity will be assessed by using a 100-mm VAS scale, with 0 = no pain and 100 = worst possible pain. Nausea will be measured by a VAS scale, with 0 = none and 100 = vomiting. Perceptual change will be assessed in five categories (i.e., body, surroundings, time, reality, colors, and sounds) by using a VAS scale anchored by “normal” at one end and “extremely” at the other end. Mood states are ranked between 0 and 100 in five categories: anxious/composed, hostile/agreeable, depressed/elated, tired/energetic, and confused/clearheaded) (modified from Mortero et al. [9]).

The day after the procedure patients are contacted by telephone to assess post-procedural satisfaction. The patient is asked the same questions from part one and two of the patient questionnaire. Patients are also be interviewed about their total satisfaction with the procedure, about their physical activity level using a 5-point rating scale: 1 = chair bound; 2 = minimal (i.e. can go to the bathroom); 3 = moderate (i.e. can go around the house and garden); 4 = almost normal; and 5 = normal, and they are asked if they would recommend this sedation regime to one of their friends. Endoscopists have to fill in questionnaires on their estimation of pain, sedation, ease of performance, and satisfaction with the procedure.

Pulmonary and cardiovascular vital signs are electronically recorded throughout the procedure and include SpO_2_ measured by pulse oximetry, etCO_2_, RR, HR, arrhythmias, and NIBP. Sedation-related pulmonary and cardiovascular incidents are defined according to the International Sedation Task Force of the World Society of Intravenous Anaesthesia (SIVA) consensus statement for standardized definitions and terminology for sedation-related adverse events [10]. Pulmonary incidents are defined as oxygen desaturation (SpO_2_ 75–90%) for < 60 s, severe (SpO_2_ < 75% at any time) or prolonged (SpO_2_ < 90% for >60 s) oxygen desaturation, apnea, prolonged apnea (>60 s), airway obstruction with need for airway interventions: facemask ventilation, guedel, nasopharyngeal airway, and endotracheal tube. Cardiovascular incidents are defined as bradycardia*, tachycardia*, hypotension*, hypertension* (*as a change >25% from baseline and/or necessitating an intervention), cardiovascular collapse and arrest. In addition, the use of atropine, ephedrine or phenylephrine intravenously to treat hypotension or bradycardia is noted.

### Statistical analysis

Statistical analyses will be performed using SPSS statistics. All data will be checked for normal distribution using the Kolmogorov test. For normally distributed data, continuous variables will be analyzed using the independent Student’s *t* test and the variables will be presented as mean ± standard deviation (SD). Non-normally distributed data will be compared using the Mann-Whitney *U* test where appropriate, and data will be presented as the median and interquartile range (IQR). For categorical variables, cross-tabulation and the Pearson chi-squared test will be applied and variables will be allegorized as number and/or percentage of the total. To compare the continuous measurements of HR, NIPD, and SpO_2_ between the groups, the area under the curve (AUC) for each value will be calculated over the different measurement time points during the procedure. A *p* value <0.05 will be considered statistically significant. Confidence intervals will be reported where appropriate.

### Ethical approval

This trial is conducted in accordance with the protocol and in compliance with the moral, ethical, and scientific principles governing clinical research as set out in the Declaration of Helsinki (1989) and Good Clinical Practice (GCP). It is registered in the Nederland’s Trial Register (NTR5486) (registration date 17 September 2015). A centralized ethics committee (the Medical Ethics Committee of the AMC, Amsterdam, The Netherlands (NL)) has approved this study for both participating centers: the AMC, Amsterdam and the Tjongerschans ziekenhuis, Heerenveen, The Netherlands. The National Authority, the Central Committee on Research Involving Human Subjects (CCMO), performed a marginal review and there were no objections to perform this study (NL53999.018.15 BI).

## Discussion

In recent years, the combination of propofol with an opioid for sedation has replaced the conventionally used combination with benzodiazepines, and became the standard for analgo-sedation during ERCP, with the advantage of improved titration of sedation, shorter recovery time, and better patient tolerance and satisfaction. However, a possible consequence of high-dose propofol sedation is that it may result in progression from deep sedation to general anesthesia. Cote et al. showed that hypoxemia occurred in 12.8% of 799 patients sedated for endoscopic procedures with propofol applied by trained anesthesia nurses [11]. Minimizing these risks is therefore an important goal to make sedation procedures safer. A possible approach is to reduce the propofol dosage using a combination with other substances.

Esketamine offers the advantages of minimizing sedation side effects, making optimal use of the concept of synergy while being an analgesic at the same time. Despite its effective anesthetic and analgesic effects, spontaneous breathing and airway reflexes are maintained and hypotension is less common due to an increase in sympathetic tone. Varadarajulu et al. [12] demonstrated that the use of ketamine in patients who were difficult to sedate during ERCP resulted in better quality of sedation and analgesia. They observed shorter recovery times compared with opiate and benzodiazepine sedation. However, they concluded that it is necessary to conduct further randomized trials. Wehrmann et al. [13] recommended the combination of propofol with ketamine because of its analgesic properties without cardiorespiratory depressant effects.

Mortero et al. [9] found that the combination of propofol with small doses of ketamine during monitored anesthesia for surgical interventions reduced hypoventilation caused by propofol, induced a stable positive spirit, and provided earlier recovery of perception in comparison to propofol alone.

The most widespread doubts about esketamine, however, correspond to its mind-altering effects in cognition. It can produce psychotomimetic effects that may be associated with symptoms similar to dissociative states of mind [14]. On the other hand, Nakao et al. [15] showed that propofol used in clinical relevant dosages suppresses these effects via the activation of GABA receptors. Unfortunately, there are only a few studies with only limited significance investigating the effectiveness of a propofol/esketamine regime with emphasis on the aforementioned safety aspects during ERCP and the eventually psychotomimetic effects such as visual disturbances, vertigo, or nausea that could compromise patient satisfaction and recovery after discharge home.

A limitation of our study could be that we based sample size calculation on the reduction of the total dosage of propofol. Probably, acute respiratory or hemodynamic adverse events would have been a more appropriate primary outcome. However, much larger-scale studies would have been necessary to address this outcome. Alongside this we defined cardiorespiratory events according to the SIVA consensus statement for standardized definitions and terminology for sedation-related adverse events. However, their clinical impact cannot be determined when a sedation specialist provides adequate rescue maneuvers during such events.

The aim of our trial is to show that the synergy of esketamine and propofol reduces the dosage of individual drugs, thereby providing a better safety and satisfaction profile than the combination with an opioid during ERCP.

## Trial status

The first patient was included on 8 December 2015. We expect to finalize the study in December 2017.

## Additional file

Additional file 1: SPIRIT checklist. (DOC 121 kb)

## Abbreviations

AMC: Academic Medical Centre
ASA: American Society of Anesthesiologists
AUC: Area under the curve
CCMO: Central Committee on Research Involving Human Subjects
CRU: Clinical Research Unit
ECG: Electrocardiogram
ERCP: Endoscopic retrograde cholangiopancreatography
EtCO2: End-tidal CO_2_
GCP: Good Clinical Practice
HR: Heart rate
IQR: Interquartile range
MOAA/S: Modified Observer’s Assessment of Alertness/Sedation Scale
NIBP: Non-invasive blood pressure
NRS: Numeric rating score
NTR: Nederland’s Trial Register
SD: Standard deviation
SIVA: Society of Intravenous Anaesthesia
SO_2_: Oxygen saturation
SPIRIT: Standard Protocol Items: Recommendations for Interventional Trials
TCI: Target controlled infusion
VAS: Visual analog scale
